# Overcoming Racial and Ethnic Disparities in Rectal Cancer Treatment

**DOI:** 10.1001/jamanetworkopen.2024.0018

**Published:** 2024-02-05

**Authors:** Cassandra D. L. Fritz, Oluseye Oduyale, Yin Cao

**Affiliations:** Division of Gastroenterology, Department of Medicine, Washington University School of Medicine, St Louis, Missouri (Fritz, Cao); Alvin J. Siteman Cancer Center, Washington University School of Medicine, St Louis, Missouri (Fritz, Cao); Editorial Fellow, *JAMA Network Open* (Fritz); Department of Surgery, Washington University School of Medicine, St Louis, Missouri (Oduyale, Cao).

Colorectal cancer (CRC) is one of the leading causes of cancer-related mortality in the US,^1^ with persistent disparities by race and ethnicity.^2^ Non-Hispanic Black individuals have some of the highest rates of CRC incidence and mortality. Stage at diagnosis is thought to be a significant contributor, with non-Hispanic Black individuals being the most likely of all racial and ethnic groups to be diagnosed with metastatic CRC (25% vs 21% of non-Hispanic White individuals).^2^ Several potential etiologies contribute to the survival disparity between non-Hispanic Black and non-Hispanic White individuals, including differing prevalences of comorbidity and unfavorable tumor characteristics (ie, location and grade). Additionally, differences in health insurance coverage account for more than 50% of the excess risk of death from CRC in non-Hispanic Black patients compared with non-Hispanic White patients,^3^ underscoring the complexity of CRC outcomes by race and ethnicity and socioeconomic status.

Shulman and colleagues^4^ evaluated rates of pathologic complete response for locally advanced rectal cancer (stage II or III) by investigating whether social determinants of health (SDOH; including educational level, income, marital status, geographic location, and access to treatment), along with other demographic, clinical, or pathologic factors, mediate racial and ethnic disparities. The authors performed a retrospective cohort study using the National Cancer Database from 2004 to 2017, including 34 500 patients (85.3% non-Hispanic White, 8.2% non-Hispanic Black, and 6.4% Hispanic). Compared with non-Hispanic White patients, non-Hispanic Black and Hispanic patients had notable differences, including younger age, lower education and income levels, lack of private insurance, and greater likelihood of obtaining care in low-volume treatment centers. Hispanic and non-Hispanic Black patients had greater nodal involvement and higher tumor stage than non-Hispanic White patients, without a difference in tumor grade between Hispanic and non-Hispanic Black patients. However, only non-Hispanic Black race and ethnicity emerged as an independent risk factor associated with achieving less frequent pathologic complete response compared with non-Hispanic White patients for locally advanced rectal cancer.

With these findings, the authors provide some clarity to the puzzling landscape of inequities in rectal cancer care. Intervening on inequities within locally advanced rectal cancer first requires clearly defined outcomes of interest given the heterogeneity of locally advanced rectal cancer and the multiple accepted treatment modalities, each with associated risks and benefits.^5^ Sphincter preservation, survival, and even avoidance of harmful treatment, such as surgery or radiation, are important outcomes to consider. In this study, Shulman et al^4^ explore factors associated with pathologic response, but certain questions remain unanswered. Total neoadjuvant therapy is associated with decreased toxic effects but improved tumor response, but it is unspecified in this study which patients were recipients.^6^ Furthermore, patients with a total radiation dose of less than 40 Gy were excluded, thus missing the opportunity to investigate outcomes in patients who received short-course radiation therapy. Notably, short-course radiation therapy has been found to reduce treatment time and toxicity, leading to a higher rate of therapy completion in all patients,^7^ while total neoadjuvant therapy is associated with higher rates of completion of chemotherapy among patients who postoperatively may be unable to tolerate chemotherapy or those who are lost to follow-up.^6^ These treatment modalities, in conjunction with improving access to high-volume academic centers,^8^ offer avenues through which disparities in locally advanced rectal cancer may be mitigated.

The authors also highlight that non-Hispanic Black and Hispanic patients presented with CRC at significantly younger ages and were more likely to have advanced-stage disease compared with non-Hispanic White patients, potentially contributing to downstream racial and ethnic disparities. Future work in epigenomic research will hopefully expand our understanding of the role that differing environments and behaviors may play within and between racial and ethnic categories and how these changes potentially contribute to racial and ethnic disparities. DNA methylation is only one part of the epigenomic signature, but research has begun to highlight potential colonic methylation differences by race and ethnicity, age, and location.^9,10^

We must also acknowledge inadequacies in current research. It is difficult to capture, then detangle the numerous interacting factors associated with disparities in cancer care. As seen in this study, cancer registries often lack granular data on treatment modalities and rely on zip-code–level data to identify social determinants of health, including income and education. Consequently, these studies often fall short in their explanations of systemic obstacles that members of racial and ethnic minority groups face in pursuing CRC-related care. The authors’ work highlights what many know to be true: non-Hispanic Black patients face different levels of health care discrimination than members of other racial and ethnic groups. Of the 5 categories included in social determinants of health (education, health care quality and access, neighborhood environment, social and community context, and economic stability), social and community context, including stress and racial and ethnic discrimination, is likely the most difficult to account for in studies. However, when at least 1 SDOH factor is included to adjust for almost all other SDOH categories, we need to draw conclusions that clearly state that systemic racism and racial and ethnic discrimination in health care are contributing to outcomes.

Black patients experience present-day and persistent, not just historic, mistreatment within the health care system. Naming Black “mistrust” of the health care system and “perceived discrimination” as factors that shape outcomes for non-Hispanic Black patients is troubling and incorrect. Patient trust will not improve outcomes by race and ethnicity, but addressing racism and other prejudice in health care and bias among health care professionals may solve racial and ethnic health inequities.^11,12^ Furthermore, like many other studies, this one offers a potential genetic explanation for disparate racial and ethnic outcomes, an approach that is unsubstantiated.^12^ Ultimately, race and ethnicity are social constructs that we must take care not to conflate with underlying genetic differences.

Overall, the examination of racial and ethnic disparities in locally advanced rectal cancer by Shulman et al^4^ sheds light on the complex interplay of SDOH and clinical factors that contribute to divergent outcomes. The study underscores the urgency of addressing disparities in cancer care, particularly for non-Hispanic Black patients, who face distinct challenges. While we see valuable insights into factors associated with pathologic response, unanswered questions persist, necessitating further research within diverse communities. In recognizing limitations of current research on the multifaceted nature of health care disparities, it is imperative to clearly acknowledge the impact of systemic racism. Moving forward, interventions aimed at mitigating disparities in locally advanced rectal cancer must focus on equitable prevention strategies and improving access to treatment modalities, while prioritizing the dismantling of systemic barriers that ultimately transcend boundaries of race and ethnicity as social constructs.
